# Compound Heterozygosity for *KLF1* Mutations Causing Hemolytic Anemia in Children: A Case Report and Literature Review

**DOI:** 10.3389/fgene.2021.691461

**Published:** 2021-06-25

**Authors:** Linlin Xu, Dina Zhu, Yanxia Zhang, Guanxia Liang, Min Liang, Xiaofeng Wei, Xiaoqing Feng, Xuedong Wu, Xuan Shang

**Affiliations:** ^1^Department of Medical Genetics, School of Basic Medical Sciences, Southern Medical University, Guangzhou, China; ^2^Department of Pediatrics, Nanfang Hospital, Southern Medical University, Guangzhou, China

**Keywords:** KLF1, compound heterozygote, hereditary hemolytic anemia, children, chronic non-spherocytic hemolytic anemia, β-thalassemia

## Abstract

**Background:**

Anemia is one of the most common diseases affecting children worldwide. Hereditary forms of anemia due to gene mutations are difficult to diagnose because they only rely on clinical manifestations. In regions with high prevalence of thalassemia such as southern China, pediatric patients with a hereditary hemolytic anemia (HHA) phenotype are often diagnosed with β-thalassemia. However, HHA can be caused by other gene defects. Here, a case previously diagnosed with thalassemia in a local hospital was sent to our laboratory for further genetic diagnosis. Preliminary molecular testing did not identify any mutations in globin genes.

**Methods:**

All blood samples were collected after informed consent had been obtain from the proband’s parents. Both clinical and genetic analyses were conducted for the patient and her family members, including clinical data collection and sequencing of the *KLF1* gene. Relevant literature was reviewed, including genetically confirmed cases with well-documented clinical summaries.

**Results:**

Based on the detailed clinical data for this case, we diagnosed the patient with severe HHA. Sanger sequencing confirmed that there was a mutation on each KLF1 allele in the proband, which is missense mutation c.892G > C (p.Ala298Pro) inherited from father and frameshift mutation c.525_526insCGGCGCC (p.Gly176Argfs^∗^179) from the mother, respectively. A summary of the *KLF1* mutation spectrum and a clarification of genotype–phenotype correlation were performed through a combined analysis of the case and literature studies.

**Conclusion:**

This study corrected the misdiagnosis and identified the etiology in a Chinese patient with HHA. Identification of the disease-causing gene is important for the treatment and care of the patient and prevention of another affected childbirth in her family. In addition, this study provided insight to better distinguish HHA patients with β-thalassemia mutations from those with *KLF1* mutations.

## Introduction

Anemia is the most common blood disease worldwide (Kassebaum et al., 2014), which is defined by a hemoglobin (Hb) level below the normal value, leading to decreased oxygen-carrying capacity. Severe anemia impairs growth, cardiac function, and cognitive development in infants. As such, anemia is a leading cause of morbidity and mortality in children in tropical countries (Reyburn, 2010; Khan, 2018).

Anemia is often multifactorial, including genetic and environment factors. Although nutritional-deficiency anemia, due to iron deficiency, is typically assumed to be the most frequent cause in children (Allali et al., 2017), hereditary forms of anemia caused by mutations are not uncommon in the clinic. Moreover, patients with hereditary anemia are often concerned by pediatricians because they are difficult to diagnose and cannot be cured completely with conventional treatment. For such patients, especially those who depend on regular transfusions, a better understanding of etiology and more accurate diagnostic methods are crucial for subsequent treatment and care.

Hereditary hemolytic anemia (HHA) is a group of diseases that result from the destruction of red blood cells (RBCs), and hemolytic anemia shows varied clinical and molecular heterogeneity (Iolascon et al., 2009; Kim et al., 2017). The typical clinical presentation includes pallor, anemia, jaundice, and often enlargement of the liver and spleen. Laboratory results that help confirm HHA include elevated reticulocyte count, increased lactate dehydrogenase, elevated unconjugated bilirubin, and decreased haptoglobin. In southern China, most pediatric HHA cases are caused by β-thalassemia, which is highly prevalent in this region (Shang et al., 2017). β-thalassemia is caused by mutations in the β-globin gene, which results in impaired Hb synthesis (Shang and Xu, 2017). Hematological phenotype analysis, combined with molecular testing for the β-globin gene, provides an avenue for accurate diagnosis. However, HHA can be caused by mutations in other genes. For example, a case of HHA in a Chinese patient was originally misdiagnosed as thalassemia and ultimately diagnosed as hereditary spherocytosis because of mutations in the ankyrin 1 (*ANK1*) gene (Zhu et al., 2020). More notably, compound heterozygous mutations in the Kruppel-like factor 1 (KLF1) gene, an important erythroid transcriptional factor involved in erythrocyte development, can lead to chronic non-spherocytic hemolytic anemia (CNSHA). The phenotypic traits of CNSHA are very similar to those of β-thalassemia (Viprakasit et al., 2014; Huang et al., 2015; Perkins et al., 2016). Because *KLF1* mutations are found significantly more frequently in thalassemia endemic regions such as southern China (Liu et al., 2014), it is important for physicians in these regions to distinguish between HHA caused by β-thalassemia from HHA caused by *KLF1* mutations.

Here, we report the case of a Chinese patient with transfusion-dependent hemolytic anemia. The patient was originally misdiagnosed with β-thalassemia by local physicians and was finally diagnosed with CNSHA caused by compound *KLF1* mutations. We also present a summary of relevant literature on the mutation spectrum of the *KLF1* gene. Moreover, to help clinicians better recognize HHA caused by *KLF1* compound heterozygotes, we compared hematological parameters in patients with β-thalassemia and *KLF1* mutations.

## Materials and Methods

### Subject

**FIGURE 1:**
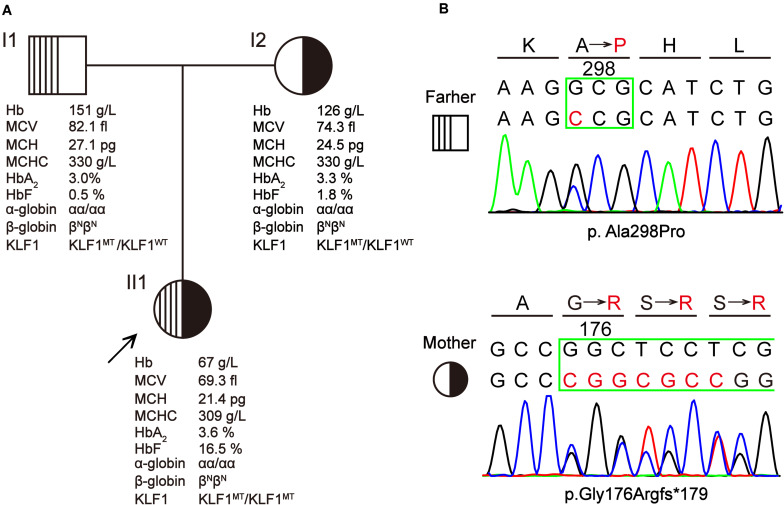
A Chinese family with *KLF1* compound heterozygote mutation causing transfusion-dependent hemolytic anemia. **(A)** The pedigree, hematological parameters, and genotype data of family members. **(B)** Sanger sequencing of the *KLF1* gene shows the p.Ala298Pro and p.Gly176Argfs*179 mutations. Amino acids are indicated above each chromatogram, with base pair and amino acid changes highlighted in red.

The proband was a 3-year-old girl from Yunfu City in Guangdong Province, southern China. She and her parents were experiencing problems with clinical diagnosis and were referred to a free clinic held by a hospital for “World Thalassemia Day—May 8.” The patient had previously experienced hyperbilirubinemia and severe anemia, and she had received eight blood transfusions before age 3 years. A local hospital initially assumed that she had β-thalassemia because she had microcytic hypochromic anemia and high HbF levels (>5%). Furthermore, she was a native of Guangdong province, which is a region with high prevalence of thalassemia. However, following molecular testing for her and her parents, no known thalassemia mutation was detected. Therefore, the patient was referred to our laboratory for further investigation. Blood samples were collected from the patient and her parents after obtaining informed consent. The pedigree of this family is shown in Figure 1A).

### Phenotype Analysis

Fresh peripheral blood samples were collected. The patient had not received a blood transfusion in the 2 months prior to sample collection. The hematological data were analyzed using an automated blood cell counter (Sysmex KX-21; Sysmex Corp., Kobe, Japan). The levels of HbA, HbA_2_, and HbF were analyzed using the Bio-Rad Variant II HPLC system (HPLC, VARIANT^TM^, Bio-Rad Laboratories, Hercules, CA, United States). Red cell osmotic fragility test, glucose-6-phosphate dehydrogenase (G6PD) activity, and other immune hemolysis test were performed according to standard operation procedure. Routine biochemical and hemolytic parameters were measured using standard techniques.

### Molecular Analysis

Sanger sequencing was used to detect point mutations in the α-globin, β-globin, and *KLF1* genes, as previously described (Zhu et al., 2019). Polymerase chain reaction (PCR) fragment were sequenced with BigDye^TM^ Terminators V3.1 Ready Reaction Kit using 3130 Genetic Analyzer (Applied Biosystems). Reference sequences were downloaded from NCBI database^1^ and used for sequence alignment and analysis. The multiplex ligation-dependent probe amplification assay (MLPA; SALSA P140-C1 HBA or P140-B2 HBB; MRC-Holland, Amsterdam, the Netherlands) was performed to exclude large rearrangements of the α-/β-globin gene clusters.

### Literature Review

An electronic literature search of the PubMed database was performed for current and past findings of the molecular genetics of *KLF1* from the years 1990 to 2020. The following keywords were used to search: “Kruppel-like factor 1,” “*KLF1*,” “EKLF,” “hemolytic anemia,” “mutation,” and “variant.” All related publications were reviewed to identify other relevant articles that were not identified in the initial research. The publications were limited to English-language articles. Cases with detailed clinical presentation and *KLF1* genotype information were selected.

### Statistical Analysis

Statistical differences between groups were determined by one-way analysis of variance, and further comparison across multiple groups was tested by Tukey method. Mean and standard error of the mean were used to describe clinical data in each group. Statistical significance was determined by a *P*-value of less than 0.05. All statistical analyses were performed using the statistical software GraphPad Prism 6.

## Results

### Hematological Evaluation and Molecular Analysis of the Patient

Hematological data for this family are summarized in Figure 1A. The father displayed a normal phenotype, except for borderline mean corpuscular Hb (MCH) and HbF level. The mother displayed microcytic and hypochromic features, as well as a slightly elevated HbF level. Patient II1 presented with severe microcytic hypochromic anemia, borderline HbA_2_ (3.6%) and significantly elevated HbF (16.5%); she has required regular transfusions for survival. This patient was born at full term (38 weeks + 2 days’ gestation) with a birth weight of 2.740 kg. The day after birth, she developed severe jaundice and neonatal hyperbilirubinemia (total and indirect bilirubin were 133.4 and 109.9 μmol/L, respectively), for which she received phototherapy and was referred to a superior hospital. She was followed up as a high-risk baby, at which point she was found to be anemic with hepatosplenomegaly (4 and 3 cm, respectively); she received her first blood transfusion within 1 month after birth. Peripheral blood smear analysis showed increased poikilocytosis and anisocytosis, with hypochromic microcytosis, similar to what is observed with thalassemia. The patient has required regular blood transfusion every 3 months since 4 months of age to keep Hb > 90 g/L, although the interval of blood transfusions was reduced during times when the patient experienced fever or infection. Routine evaluations excluded the possibility of immune hemolysis and RBC membrane disorder. At a local hospital, she was initially diagnosed with β-thalassemia by age 1 year based on her abnormal Hb profiles and the hematological features of microcytic hypochromic anemia. The routine test for common β-thalassemia mutations in Chinese patients by reverse dot blot and gap-PCR methods showed a negative result. Therefore, the patient was thought to have hereditary anemia due to an unidentified thalassemia allele (possible *de novo* mutation), and she was referred to our laboratory for further study.

We have provided an in-depth review of the patient’s hematological parameters in (Table 1). Complete blood cell analysis showed white blood cell, and platelet values were within normal levels. However, RBC parameters suggested moderate anemia with hypochromic microcytosis. Elevated reticulocyte count and red cell osmotic fragility indicated the existence of hemolysis. Immune hemolysis and erythrocyte enzymatic disease (G6PD deficiency) were excluded by related hemolysis tests. Biochemical tests suggested mild damage to liver function with increased levels of alanine aminotransferase, aspartate aminotransferase, and alkaline phosphatase. Elevated bilirubin and decreased globulins further confirmed hemolysis and jaundice. Iron deficiency and megaloblastic anemia were excluded by the normal values of ferritin, folic acid, and vitamin B_12_, respectively. Therefore, the patient was ultimately diagnosed with non-spherocytic hemolytic anemia.

**TABLE 1:** Current, in-depth hematological parameters of subject II1.

**White blood cell parameters**	**Relative values (%)**	**Reference values (%)**	**Absolute values (10^6^/μL)**	**Ref. values (10^6/^μL)**
WBC			10.24 ↑	4.00–10.00
NEU^a^	59.9	50.0–70.0	6.14	2.00–7.00
LYM	31	20.0–40.0	3.17	0.80–4.00
MON	5.9	3.0–12.0	0.6	0.12–1.20
EOS	2.7	0.5–5.0	0.28	0.02–0.50
BAS	0.5	0.0–1.0	0.05	0.00–0.10

**Platelet parameters**	**Values**	**Reference values**		

PLT^a^ (×10^9^/L)	192	125–350		
PCT (%)	0.14	0.05–0.33		

**Red blood cell parameters**	**Values**	**Reference values**		

RBC (10^12^/L)	3.13 ↓	4.0–5.5		
Hb^a^ (g/L)	67 ↓^c^	110–140		
HCT (%)	21.7 ↓	35.0–45.0		
MCV (fL)	69.3 ↓	82.0–100.0		
MCH (pg)	21.4 ↓	27.0–34.0		
MCHC (g/L)	309 ↓	316–354		
RDW-CV (%)	26.5 ↑	11.5–14.5		

**Reticulocyte parameters**	**Relative values (%)**	**Reference values (%)**	**Absolute values (10^6^/μL)**	**Ref. values (10^6^/μL)**

RET	16.1 ↑^c^	0.5–2.1	566.8 ↑	40.0–79.0

**Hemolysis test**	**Values**	**Reference values**		

Isopropanol test	–	–		
Heinz-bodyforming test	–	–^b^		
Red cell osmotic fragility test	–	–		
G6PD activity test	+	+		
Ham test	–	–		
Sucrose hemolysis test	–	–		
Heat hemolysis test	–	–		
Direct Coombs test	–	–		
Indirect Coombs test	–	–		

**Biochemical tests**	**Values**	**Reference values**		

Uric Acid (UA; umol/L)	224	120–420		
Creatinine (Cre; umol/L)	46	40–88		
Total Protein (TP; g/L)	56.4 ↓	60–80		
Albumins (ALB; g/L)	42.3	35–55		
Globulins (GLB; g/L)	14.1 ↓	20–30		
Total bilirubin (TBIL; umol/L)	28.4 ↑	5.1–19		
Direct bilirubin (DBIL; umol/L)	6.4	1.7–6.8		
Indirect bilirubin (IBIL; umol/L)	22.0 ↑^c^	0.0–17.3		
Alkaline phosphatase (ALP; U/L)	236 ↑	42–98		
γ-glutamyl transferase (γ-GT; U/L)	11	7–50		
Alanine aminotransferase (ALT; U/L)	36 ↑	5–35		
Aspartate aminotransferase (AST; U/L)	76 ↑^c^	8–40		
C-reactive protein (CRP; mg/L)	2.5	0.0–10.0		
Ferritin (FER; pmol/L)	277.5	22.0–610		
Folic Acid (FA; nmol/L)	21	>12.9		
Vitamin B12 (VB12; pmol/L)	230	156–672		

*^a^WBC, white blood cell; NEU, neutrophil; LYM, lymphocyte; MON, monocyte; EOS, eosinophil; BAS, basophil; PLT, platelet count; RBC, red blood cell; Hb, hemoglobin; HCT, hematocrit; MCV, mean corpuscular volume; MCH, mean corpuscular; MCHC, mean corpuscular hemoglobin concentration; RDW-CV, red cell distribution width.*

*^b^– indicate negative phenotypes described in rows.*

*^c^↑ and ↓ indicate increase and decrease expression.*

Based on hematological data from the parents (Figure 1A), it is likely that the patient inherited a recessive disease. However, mutations in the α-, β-, or γ-globin genes that could cause thalassemic phenotypes similar to those seen in the patient were excluded by direct sequencing of above genes and MLPA analysis of α- or β-globin cluster. This indicated that there might be another gene responsible for the observed hemolytic anemia phenotypes. Considering the high HbF levels in the patient, *KLF1* was chosen to be the first candidate gene (Borg et al., 2010). Sequencing of the *KLF1* gene identified a compound heterozygote of missense variant c.892G > C (p.Ala298Pro) and frameshift variant c.525_526insCGGCGCC (p.Gly176Argfs^∗^179) in the patient, which she inherited from her father and mother, respectively (Figure 1B). Both variants have been previously reported as pathogenic mutations that affect the biological function of KLF1 (Viprakasit et al., 2014; Huang et al., 2015).

### Literature Review of *KLF1* Mutation Spectrum and Genotype–Phenotype Correlation in Individuals With *KLF1* Mutations

*KLF1* (OMIM# 600599) is located at chromosome 19p13.2 and contains three exons that encode 362 amino acids. There are two major functional domains in the KLF1 protein product: two PRA (proline-rich amino) domains located at the N-terminus function as a transcriptional activator and three zinc finger (ZF) domains located on the C-terminus act as a DNA-binding domain (Quadrini and Bieker, 2002).

**FIGURE 2:**
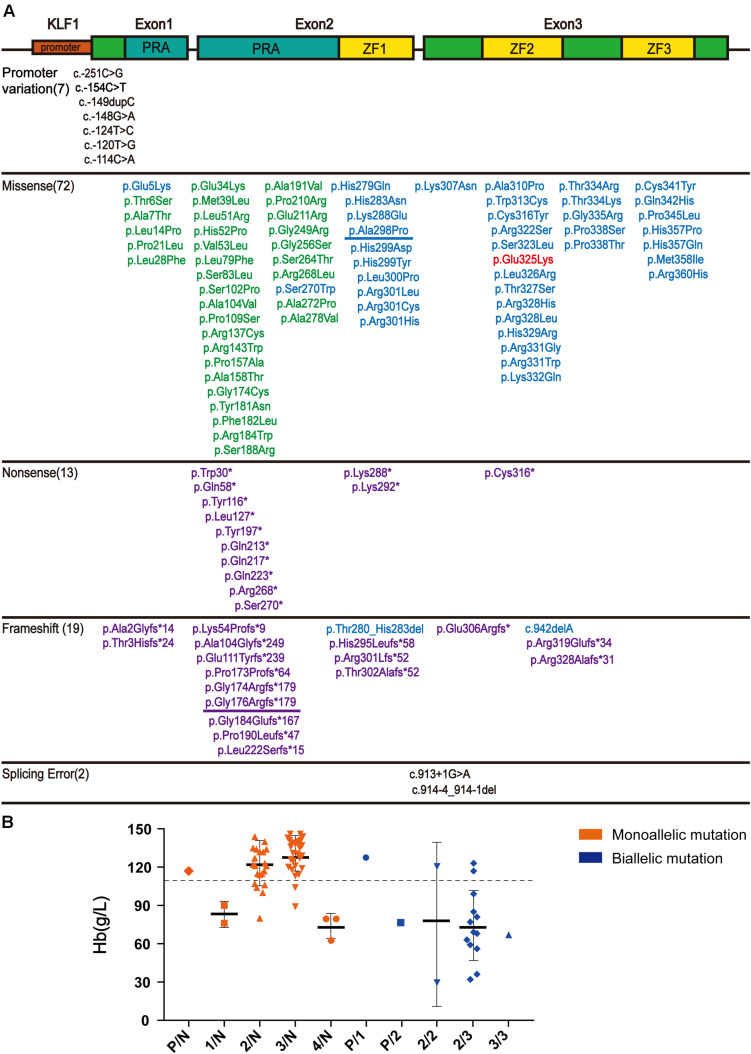
The literature review of *KLF1* mutations and phenotype. **(A)** Schematic diagram of the *KLF1* gene (NM_006563.5 and NP_006554.1) structure and variants, as reported in the literature. The gene structure includes the promoter (small red box) and two introns and three exons (big box). The proline-rich amino domain (PRA) located at the N-terminus is responsible for transcriptional activation (blue box). The zinc finger (ZF) domains (yellow box) located at C-terminus enable KLF1 to bind specific sites on DNA. Mutations are color-coded: promoter mutations and splicing mutations, black; class 1, green; class 2, blue; class 3, purple; class 4, red. The two mutations described in this article are underlined. See Supplementary Table 1 for details and references. **(B)** Hb level in reported cases carrying *KLF1* mutations. All *KLF1* alleles were classified into six types: N, normal allele; P, promoter mutations; 1–4, classes 1–4 mutations. The dotted line shows the anemia boundary line. See Supplementary Table 2 for details and references.

After searching the literature for *KLF1* mutations, 113 mutations were identified (Supplementary Table 1). These mutations can be divided into three groups according to their position on *KLF1* gene. Seven mutations are located on the promoter region, and therefore may affect transcription of the *KLF1* gene. Additionally, 104 mutations (72 missense mutations, 13 nonsense mutations, and 19 frameshift mutations) are located on the exon region and cause alterations to the primary structure of the KLF1 protein. Although the ZF domains comprise only 20% of KLF1, almost 40% of the functionally effective mutations are found within and between these domains. Two intronic mutations affect splicing. In addition, the 104 mutations in the coding sequence (CDS) region can be divided into four subclasses, based primarily on differing effects on KLF1 protein function (Figure 2A). Class 1 includes 33 mutations with no effects or minor functional effects, most of which are located outside the ZF domains. Class 2 comprises 40 mutations, most of which are missense mutations in ZF domains that interfere with normal function of KLF1. Class 3 includes 30 nonsense or frameshift mutations, which lead to a truncated KLF1 peptide that lacks all or part of the ZF domains. Class 4 contains only one missense mutation (p.Glu325Lys) (Arnaud et al., 2010), which is a special dominant variant.

According to published literature, 80 cases with detailed hematological phenotype data and *KLF1* genotype were collected (Supplementary Table 2). We have presented the genotype–phenotype relationship in Figure 2B. Most cases carrying monoallelic *KLF1* mutations do not display anemia. On the contrary, most cases carrying biallelic mutations do exhibit an anemia phenotype, as their Hb level is often less than 110 g/L.

## Discussion

HHA includes a diverse group of genetically and phenotypically heterogeneous diseases. The major causes of HHA are defects in Hb, the RBC membrane, and RBC enzymes, which are commonly referred to as thalassemia, hereditary spherocytosis, and G6PD deficiency, respectively. However, these common disorders do not represent all HHA patients, and precise diagnosis of HHA remains a challenge for clinical doctors. As clinical presentation often overlaps in cases with different etiologies, it is difficult to make a reliable diagnosis based only on phenotype analysis. Identification of disease-causing mutations at the DNA level can increase the accuracy of HHA diagnosis. Our study provided a new cause of HHA in pediatric patients, *KLF1* compound mutations. The patient discussed here had jaundice, hepatomegaly, and microcytic hypochromic anemia with remarkably elevated HbF levels.

KLF1 is known as a hematopoietic-specific transcription factor that regulates erythroid lineage commitment, globin switching, and the terminal maturation of RBCs (Miller and Bieker, 1993; Wijgerde et al., 1996; Hariharan et al., 2019). It is no wonder that *KLF1* mutations can lead to shortened survival of RBCs, resulting in inherited hemolytic anemias. Currently, there are reports of 19 compound heterozygotes of *KLF1* mutations (including our case) with detailed hematological phenotypes (Table 2). Four cases (Table 2, numbers 1–4) showed normal Hb levels with high HbF and were described as hereditary persistence of fetal Hb (HPFH) (Satta et al., 2011; Radmilovic et al., 2013; Rani et al., 2018), a kind of benign hemoglobinopathy. Fifteen cases (Table 2, numbers 5–19) showed mild to severe anemia (Viprakasit et al., 2014; Huang et al., 2015; Magor et al., 2015; Lee et al., 2016; Jiang et al., 2019; Belgemen-Ozer and Gorukmez, 2020). Although some cases carry pathogenic mutations in globin genes that further affect the hematological phenotype, these cases suggest that compound heterozygotes have a high probability of showing a transfusion-dependent anemia phenotype in childhood.

**TABLE 2:** Literature reports of clinical and laboratory characteristics of compound heterozygotes for *KLF1* mutations.

**Case**	**Age- sex**	**Ethnic**	**Hb (g/L)**	**MCV (fL)**	**MCH (pg)**	**HbA_2_ (%)**	**HbF (%)**	**Transfusion dependent**	**Globin genotype**		***KLF1* genotype**		**Description**	**References**
									**α globin**	**β globin**	**Mutation 1**	**Mutation2**		
1	31y-M	Sardinia	115	82.0	27.6	2.8	30.9	No	αα/αα	NA	Ser270*	Lys332Gln	HPFH	Satta et al., 2011
2	30y-M	Sardinia	121	73.0	24.3	3.3	22.1	No	−α/αα	NA	Ser270*	Lys332Gln	HPFH	Satta et al., 2011
3	NA-F	Serbia	128	NA	22.6	2.5	11.0	No	NA	NA^a^	−148G > A	Ser102Pro	HPFH^a^	Radmilovic et al., 2013
4	56y-M	India	121	91.1	30.3	0.5	72.3	No	αα/αα	β/β	Arg301Cys	Arg301Cys	HPFH	Rani et al., 2018
5	12y-M	China	97	72.4	25.1	3.9	33.2	Yes	αα/αα	β/β	Gly176Argfs*179	Pro338Ser	CDA IV^a^	Huang et al., 2015
6	5y-F	Thailand	83	70.9	21.6	3.0	50.0	Yes	α^CS^α/αα	β/β	Gly176Argfs*179	Ala298Pro	CNSHA	Viprakasit et al., 2014
7	4y-M^c^	China	79	72.4	24.9	4.8	26.6	No	αα/αα	β/β	Gly176Argfs*179	Ala298Pro	CDA IV	Huang et al., 2015
8	1y-M	Thailand	77	73.0	23.0	22.7	52.8	Yes	αα/αα	β^E^/β^E^	–154C > T	Ala298Pro	CNSHA	Viprakasit et al., 2014
9	12y-M	Thailand	75	69.1	20.1	4.3	39.1	Yes	–^SEA^/αα	β^E^/β	Gly176Argfs*179	Arg301His	CNSHA^a^	Viprakasit et al., 2014
10	1y-M	China	67	88.9	28.5	2.0	24.6	Yes	αα/αα	β/β	Gly176Argfs*179	Pro338Thr	CNSHA	Jiang et al., 2019
11	3y-F^c^	China	67	69.3	21.4	3.2	16.5	Yes	αα/αα	β/β	Gly176Argfs*179	Ala298Pro	CNSHA	Our study
12	5y-M	Thailand	66	69.5	21.4	9.5	2.6	Yes	–^SEA^/–α^3.7^	β^E^/β	Gln58*	Ala298Pro	CNSHA	Viprakasit et al., 2014
13	6mon-M	Australia	65	82.0	NA	< 0.1	75.0	Yes	NA^a^	NA	Arg319Glufs*34	Trp30*	CNSHA	Magor et al., 2015
14	7mon-M	NA^a^	61	82.0	28.4	1.0	66.5	No	αα/αα	β/β	His295Leufs*58	Arg301Leu	CDA IV	Belgemen-Ozer and Gorukmez, 2020
15	2mon-M	Thailand	57	67.0	21.2	2.2	54.6	Yes	–α^3.7^/αα	β/β	Gly176Argfs*179	Ala298Pro	CNSHA	Viprakasit et al., 2014
16	4y-M	Thailand	54	75.0	24.7	10.0	19.0	Yes	αα/αα	β^E^/β	Gly176Argfs*179	Ala298Pro	CNSHA	Viprakasit et al., 2014
17	18y-M	Thailand	34	81.0	24.8	4.6	29.0	Yes	αα/αα	β/β	Gly176Argfs*179	Ala298Pro	CNSHA	Viprakasit et al., 2014
18	27w^b^-F	China	30	123.9	37.6	NA^a^	64.0	Yes	αα/αα	NA	Gly176Argfs*179	Pro338Thr	CDA IV	Lee et al., 2016
19	4y-F	Thailand	30	80.1	28.1	3.1	16.2	Yes	αα/αα	β/β	Gly335Arg	Arg331Trp	CNSHA	Viprakasit et al., 2014

*^*a*^NA, not available; HPFH, hereditary persistence of fetal hemoglobin; CDA IV, congenital dyserythropoietic anemia IV; CNSHA, congenital non-spherocytic hemolytic anemia.*

*^*b*^Indicates 27 week fetus during pregnancy.*

*^*c*^M and F indicates male and female, respectively.*

**FIGURE 3:**
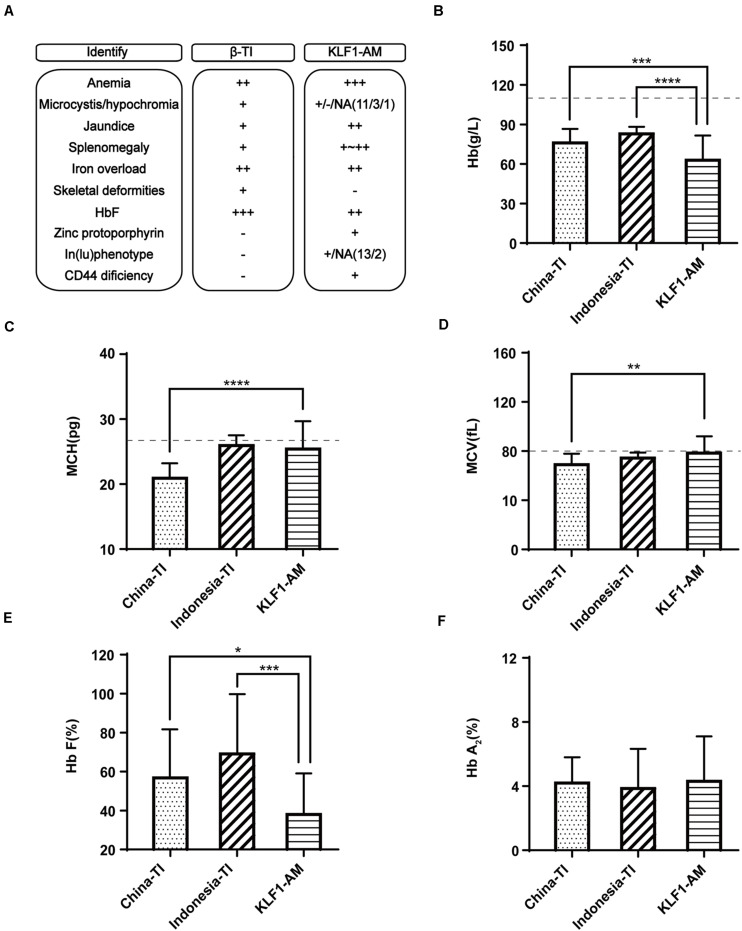
Differential diagnosis of *KLF1* anemia and β-thalassemia intermedia. **(A)** Summary of clinical characteristic features in Chinese patients with β-thalassemia intermedia (China-TI), Indonesian patients with β-thalassemia intermedia (Indonesia-TI) and anemia caused by compound heterozygotes of *KLF1* mutations (*KLF1*-AM). NA indicates not available; + and −, positive and negative phenotypes described in the columns; ++ or +++, degree of symptom. **(B–F)** Comparisons of Hb, MCV, MCH, HbF, and HbA_2_ levels between China-TI, Indonesia-TI, and *KLF1*-AM. Data are presented as mean ± standard deviation (SD). Reference range values are shown as a dotted line: MCV, 80 fL; MCH, 27 pg; China-TI, β-thalassemia intermedia patients in China; Indonesia-TI, β-thalassemia intermedia patients in Indonesia; *KLF1*-AM, cases caused by *KLF1* compound mutations. Asterisks represent statistically significant differences: *P < 0.05, **P < 0.01, ***P < 0.001, ****P < 0.0001.

Southern China is a region with a high incidence of β-thalassemia, and local pediatricians receive considerable training in thalassemia through long-term public health education and clinical practices. However, this has raised another problem in that patients in this region with a hemolytic anemia phenotype and recessive inheritance were more likely to be diagnosed with β-thalassemia. According to a previous publication (Liu et al., 2014), southern China is also a region with a high prevalence of *KLF1* mutations. Therefore, patients with *KLF1* compound mutations should account for a certain proportion of all hemolytic anemia patients. To avoid misdiagnosis, it is necessary to effectively distinguish between anemia caused by *KLF1* mutations and anemia caused by β-thalassemia mutations. According to previous reports (Huang et al., 2015), most patients with HHA caused by compound mutations of *KLF1* were from Chinese or southeast Asian populations, and they tended to be diagnosed with β-thalassemia intermedia (β-TI) at the local hospital. Here, 44 TI patients from southern China (Chen et al., 2010) along with 30 TI patients from Indonesia (Rujito et al., 2016) (reported previously) were selected for comparison with the 15 previously referenced cases that were caused by *KLF1* compound mutations (*KLF1*-AM; cases 5–19 in Table 2). A summary of typical clinical features is shown in Figure 3A. Some important symptoms were overlapping; for example, both groups presented with hypochromic microcytic anemia, jaundice, splenomegaly, severe iron overload, and elevated HbF levels. The specific symptoms [zinc protoporphyrin, In(Lu) phenotype, and CD44 deficiency] are not routine examination items in clinical practices. Therefore, it is not surprising that misdiagnosis occurs. Because whole-blood cell analysis and Hb electrophoresis are required items for anemia diagnosis and thalassemia screening, the key RBC parameters and the constitution of Hb were compared in patients with TI or *KLF1-*AM (Figures 3B–F). Hb values and HbF levels were significantly lower in *KLF1*-AM patients. Additionally, significant differences in mean corpuscular volume (MCV) and MCH values were evident between Chinese TI patients and *KLF1*-AM patients, suggesting that these two values may be useful in distinguishing *KLF1*-AM from β-TI in southern China. Patients with an HHA phenotype but borderline-low MCV and MCH values (rather than markedly decreased MCV and MCH values) are more likely to have *KLF1* mutations.

In conclusion, our study corrected the misdiagnosis and identified the true cause of disease in an HHA patient. The mutation spectrum of *KLF1* and the genotype–phenotype correlation of *KLF1* were reviewed. Furthermore, we propose that pediatricians in southern China should be cautious in making a diagnosis in children presenting with HHA. For difficult cases, further molecular testing should be performed, and current clinical and molecular reports should be reviewed to ensure that the final diagnosis is accurate.

## Data Availability Statement

The original contributions presented in the study are included in the article/Supplementary Material, further inquiries can be directed to the corresponding author/s.

## Ethics Statement

The studies involving human participants were reviewed and approved by Institutional Ethical Committee of Nanfang Hospital, Southern Medical University. Written informed consent to participate in this study was provided by the participants’ legal guardian/next of kin. Written informed consent was obtained from the individual(s), and minor(s)’ legal guardian/next of kin, for the publication of any potentially identifiable images or data included in this article.

## Author Contributions

LX performed the experiments, analyzed the data, and wrote the manuscript. DZ, YZ, and GL performed the experiments. ML and XFW provided technical support for this work. XF and XDW were responsible for accumulating data and referring patients for the laboratory investigations. XS designed the study and drafted the manuscript. All authors reviewed, edited, and approved the version to be submitted.

## Conflict of Interest

The authors declare that the research was conducted in the absence of any commercial or financial relationships that could be construed as a potential conflict of interest.

## Footnotes

1 https://www.ncbi.nlm.nih.gov/gene
