# Supplementary material for: Compound Heterozygosity for KLF1 Mutations Causing Hemolytic Anemia in Children: A Case Report and Literature Review
Source: Front Genet. 2021 Jun 25;12:691461. doi: 10.3389/fgene.2021.691461 (PMC8267787; doi:10.3389/fgene.2021.691461)
Supplement: Supplementary file 1 [file Data_Sheet_1.docx]

**Compound heterozygosity for *KLF1* mutations causing hemolytic anemia in children: A Case Report and Literature Review**

**Supplementary information**

**Supplemental Table 1.** *KLF1* mutations reported in the public domain

| **Location** | | **Protein/DNA variant** | **Class** | **Reference** | **MAF**  **(CHS)** | **SIFT** | **Poly­Phen** | **CADD** | **Mutation Assessor** |
| --- | --- | --- | --- | --- | --- | --- | --- | --- | --- |
| Promoter | | -251C>G |  | [PMID: 24930900](http://www.ncbi.nlm.nih.gov/pubmed/24930900) | 0.6 | - | - | 1 | - |
|  |  | -154C>T |  | [PMID: 24443441](http://www.ncbi.nlm.nih.gov/pubmed/24443441) | - | - | - | 9 | - |
|  |  | -149dupC |  | [PMID: 25976964](http://www.ncbi.nlm.nih.gov/pubmed/25976964) | 0 | - | - | - | - |
|  |  | -148G>A |  | [PMID: 23161389](http://www.ncbi.nlm.nih.gov/pubmed/23161389) | 0 | - | - | 1 | - |
|  |  | -124T>C |  | [PMID: 18487511](http://www.ncbi.nlm.nih.gov/pubmed/18487511) | - | - | - | 18.9 | - |
|  |  | -120T>G |  | [rs548543206](http://www.ncbi.nlm.nih.gov/snp/?term=rs548543206) | 0 | - | - | 12 | - |
|  |  | -114C>A |  | [PMID: 24443441](http://www.ncbi.nlm.nih.gov/pubmed/24443441) |  | - | - | 8.101 | - |
| Exon1 | PRA | p.Ala2Glyfs*14 | 3 | [PMID: 25976964](http://www.ncbi.nlm.nih.gov/pubmed/25976964) | - | - | - | - | - |
|  |  | p.Thr3Hisfs*24 | 3 | [PMID: 25976964](http://www.ncbi.nlm.nih.gov/pubmed/25976964) | - | - | - | - | - |
|  |  | p.Glu5Lys | 2 | [PMID: 24829204](http://www.ncbi.nlm.nih.gov/pubmed/24829204) | 0 | 0.03 | 0.338 | 17 | 0.438 |
|  |  | p.Thr6Ser | 1 | [PMID: 24829204](http://www.ncbi.nlm.nih.gov/pubmed/24829204) | - | 0.01 | 0.081 | 19 | 0.438 |
|  |  | p.Ala7Thr | 1 | [rs201164683](http://www.ncbi.nlm.nih.gov/snp/?term=rs201164683) | - | 0.21 | 0.007 | 16 | 0.314 |
|  |  | p.Leu14Pro | 1 | [PMID: 24829204](http://www.ncbi.nlm.nih.gov/pubmed/24829204) | - | 0 | 0.894 | 24 | 0.454 |
|  |  | p.Pro21Leu | 1 | [rs559879342](http://www.ncbi.nlm.nih.gov/snp/?term=rs559879342) | 0 | 0.34 | 0 | 15 | 0.245 |
|  |  | p.Leu28Phe | 1 | [rs375248069](http://www.ncbi.nlm.nih.gov/snp/?term=rs375248069) | - | 0.34 | 0.021 | 19 | 0.454 |
| Exon2 | PRA | p.Trp30* | 3 | [PMID: 25724378](http://www.ncbi.nlm.nih.gov/pubmed/25724378) | - | - | - | 42 | - |
|  |  | p.Glu34Lys | 1 | [rs368908352](http://www.ncbi.nlm.nih.gov/snp/?term=rs368908352) | - | 0.07 | 0.007 | 20 | 0.422 |
|  |  | p.Met39Leu | 1 | [PMID: 20676099](http://www.ncbi.nlm.nih.gov/pubmed/20676099) | 0 | 1 | 0 | 17 | 0.065 |
|  |  | p.Leu51Arg | 1 | [PMID: 22102705](http://www.ncbi.nlm.nih.gov/pubmed/22102705) | - | - | - | 15.25 | - |
|  |  | p.His52Pro | 1 | [rs541471724](http://www.ncbi.nlm.nih.gov/snp/?term=rs541471724) | 0 | 0.02 | 0.121 | 16 | 0.374 |
|  |  | p.Val53Leu | 1 | [rs143927401](http://www.ncbi.nlm.nih.gov/snp/?term=rs143927401) | 0 | 0.14 | 0.015 | 12 | 0.454 |
|  |  | p.Lys54Profs*9 | 3 | [PMID: 22102705](http://www.ncbi.nlm.nih.gov/pubmed/22102705) | - | - | - | - | - |
|  |  | p.Gln58* | 3 | [PMID: 24443441](http://www.ncbi.nlm.nih.gov/pubmed/24443441) | - | - | - | 26.4 | - |
|  |  | p.Leu79Fhe | 1 | [rs150150802](http://www.ncbi.nlm.nih.gov/snp/?term=rs150150802) | - | 0.17 | 0.031 | 17 | 0.28 |
|  |  | p.Ser83Leu | 1 | [rs374473630](http://www.ncbi.nlm.nih.gov/snp/?term=rs374473630) | - | 0.01 | 0.023 | 20 | 0.144 |
|  |  | p.Ser102Pro | 1 | [PMID: 21055716](http://www.ncbi.nlm.nih.gov/pubmed/21055716) | 0.681 | 1 | 0 | 8 | 0.01 |
|  |  | p.Ala104Val | 1 | [PMID: 23125034](http://www.ncbi.nlm.nih.gov/pubmed/23125034) | 0 | 0.28 | 0 | 2 | 0.325 |
|  |  | p.Ala104Glyfs*249 | 3 | [PMID: 23125034](http://www.ncbi.nlm.nih.gov/pubmed/23125034) | - | - | - | - | - |
|  |  | p.Pro109Ser | 1 | [rs117351327](http://www.ncbi.nlm.nih.gov/snp/?term=rs117351327) | 0.081 | 0.36 | 0.007 | 10 | 0.438 |
|  |  | p.Glu111Tyrfs*239 | 3 | [PMID: 25976964](http://www.ncbi.nlm.nih.gov/pubmed/25976964) | - | - | - | - | - |
|  |  | p.Tyr116* | 3 | [PMID: 25976964](http://www.ncbi.nlm.nih.gov/pubmed/25976964) | - | - | - | 33 | - |
|  |  | p.Leu127* | 3 | [PMID: 18487511](http://www.ncbi.nlm.nih.gov/pubmed/18487511) | - | - | - | 34 | - |
|  |  | p.Arg137Cys | 1 | [rs111888566](http://www.ncbi.nlm.nih.gov/snp/?term=rs111888566) | - | 0.24 | 0.003 | 18 | 0.245 |
|  |  | p.Arg143Trp | 1 | [rs576714319](http://www.ncbi.nlm.nih.gov/snp/?term=rs576714319) | 0 | 0.01 | 0.003 | 22 | 0.065 |
|  |  | p.Pro157Ala | 1 | [rs555308232](http://www.ncbi.nlm.nih.gov/snp/?term=rs555308232) | 0 | 0.17 | 0.054 | 14 | 0.065 |
|  |  | p.Ala158Thr | 1 | [PMID: 24829204](http://www.ncbi.nlm.nih.gov/pubmed/24829204) | - | 0.62 | 0 | 14 | 0.065 |
|  |  | p.Pro173Profs*64 | 3 | [ISBT 2012 p214](http://onlinelibrary.wiley.com/doi/10.1111/j.1423-0410.2012.01615_2.x/epdf) | - | - | - | - | - |
|  |  | p.Gly174Cys | 1 | [rs566095433](http://www.ncbi.nlm.nih.gov/snp/?term=rs566095433) | 0 | 0.13 | 0.014 | 11 | 0.224 |
|  |  | p.Gly174Argfs*179 | 3 | [PMID: 23125034](http://www.ncbi.nlm.nih.gov/pubmed/23125034) | - | - | - | - | - |
|  |  | p.Gly176Argfs*179 | 3 | [PMID: 24857170](http://www.ncbi.nlm.nih.gov/pubmed/24857170) | - | - | - | - | - |
|  |  | p.Tyr181Asn | 1 | [rs547785696](http://www.ncbi.nlm.nih.gov/snp/?term=rs547785696) | 0 | 0.04 | 0.031 | 19 | 0.224 |
|  |  | p.Phe182Leu | 1 | [PMID: 23465615](http://www.ncbi.nlm.nih.gov/pubmed/23465615) | 0.019 | 0.19 | 0.262 | 17 | 0.28 |
|  |  | p.Arg184Trp | 1 | [rs571697524](http://www.ncbi.nlm.nih.gov/snp/?term=rs571697524) | 0 | 0.01 | 0.63 | 22 | 0.261 |
|  |  | p.Gly184Glufs*167 | 3 | [ISBT 2012 p214](http://onlinelibrary.wiley.com/doi/10.1111/j.1423-0410.2012.01615_2.x/epdf) | - | - | - | - | - |
|  |  | p.Ser188Arg | 1 | [PMID: 23806141](http://www.ncbi.nlm.nih.gov/pubmed/23806141) | - | - | - | - | - |
|  |  | p.Pro190Leufs*47 | 3 | [PMID: 18487511](http://www.ncbi.nlm.nih.gov/pubmed/18487511) | - | - | - | - | - |
|  |  | p.Ala191Val | 1 | [rs552126408](http://www.ncbi.nlm.nih.gov/snp/?term=rs552126408) | 0 | 0.02 | 0.059 | 21 | 0.261 |
|  |  | p.Tyr197* | 3 | [PMID: 23125034](http://www.ncbi.nlm.nih.gov/pubmed/23125034) | - | - | - | 33 | - |
|  |  | p.Pro210Arg | 1 | [rs530729436](http://www.ncbi.nlm.nih.gov/snp/?term=rs530729436) | 0 | 0 | 0.928 | 23 | 0.28 |
|  |  | p.Glu211Arg | 1 | [PMID: 28369821](https://pubmed.ncbi.nlm.nih.gov/28369821/) | - | 0.01 | 0.787 | 26 | 0.28 |
|  |  | p.Gln213* | 3 | [ISBT 2012 p214](http://onlinelibrary.wiley.com/doi/10.1111/j.1423-0410.2012.01615_2.x/epdf) | - | - | - | 38 | - |
|  |  | p.Gln217* | 3 | [PMID: 27282573](https://pubmed.ncbi.nlm.nih.gov/27282573/) | - | - | - | 39 | - |
|  |  | p.Leu222Serfs*15 | 3 | [PMID: 23125034](http://www.ncbi.nlm.nih.gov/pubmed/23125034) | - | - | - | - | - |
|  |  | p.Gln223* | 3 | [PMID: 27282573](https://pubmed.ncbi.nlm.nih.gov/27282573/) | - | - | - | - | - |
|  |  | p.Gly249Arg | 1 | [rs201935177](http://www.ncbi.nlm.nih.gov/snp/?term=rs201935177) | 0 | 0.73 | 0.001 | 15 | 0.261 |
|  |  | p.Gly256Ser | 1 | [rs141784467](http://www.ncbi.nlm.nih.gov/snp/?term=rs141784467) | - | 0.34 | 0.009 | 11 | 0.261 |
|  |  | p.Ser264Thr | 1 | [PMID: 24829204](http://www.ncbi.nlm.nih.gov/pubmed/24829204) | 0 | 0.91 | 0 | 5 | 0.111 |
|  |  | p.Arg268* | 3 | [ISBT 2012 p214](http://onlinelibrary.wiley.com/doi/10.1111/j.1423-0410.2012.01615_2.x/epdf) | - | - | - | 37 | - |
|  |  | p.Arg268Leu | 1 | [rs199685739](http://www.ncbi.nlm.nih.gov/snp/?term=rs199685739) | 0 | 0 | 0.483 | 27 | 0.261 |
|  |  | p.Ser270* | 3 | [PMID: 21273267](http://www.ncbi.nlm.nih.gov/pubmed/21273267) | 0 | - | - | 38 | - |
|  |  | p.Ser270Trp | 2 | [PMID: 24829204](http://www.ncbi.nlm.nih.gov/pubmed/24829204) | 0 | 0 | 0.899 | 32 | 0.224 |
|  |  | p.Ala272Pro | 1 | [rs540601454](http://www.ncbi.nlm.nih.gov/snp/?term=rs540601454) | 0 | 0.37 | 0.009 | 22 | 0.261 |
|  |  | p.Ala278Val | 1 | [rs372605116](http://www.ncbi.nlm.nih.gov/snp/?term=rs372605116) | - | 0.17 | 0.124 | 23 | 0.28 |
|  | ZF1 | p.His279Gln | 2 | [PMID: 25976964](http://www.ncbi.nlm.nih.gov/pubmed/25976964) | - | - | - | 25.4 | - |
|  |  | p.Thr280_His283del | 2 | [PMID: 21821711](http://www.ncbi.nlm.nih.gov/pubmed/21821711) | - | - | - | - | - |
|  |  | p.His283Asn | 2 | [rs371057187](http://www.ncbi.nlm.nih.gov/snp/?term=rs371057187) | - | 0.05 | 0.018 | 16 | 0.245 |
|  |  | p.Lys288* | 3 | [PMID: 20676099](http://www.ncbi.nlm.nih.gov/pubmed/20676099) | - | - | - | 37 | - |
|  |  | p.Lys288Glu | 2 | [PMID: 23125034](http://www.ncbi.nlm.nih.gov/pubmed/23125034) | - | - | - | 26.9 | - |
|  |  | p.Lys292* | 3 | [PMID: 18487511](http://www.ncbi.nlm.nih.gov/pubmed/18487511) | - | - | - | 37 | - |
|  |  | p.His295Leufs*58 | 3 | [PMID: 32032242](https://pubmed.ncbi.nlm.nih.gov/32032242/) | - | - | - | - | - |
|  |  | p.Ala298Pro | 2 | [PMID: 24443441](http://www.ncbi.nlm.nih.gov/pubmed/24443441) | - | 0.01 | 1 | 33 | 0.397 |
|  |  | p.His299Asp | 2 | [PMID: 24829204](http://www.ncbi.nlm.nih.gov/pubmed/24829204) | - | 0.02 | 1 | 33 | 0.99 |
|  |  | p.His299Tyr | 2 | [PMID: 18487511](http://www.ncbi.nlm.nih.gov/pubmed/18487511) | - | 0 | 1 | 33 | 0.962 |
|  |  | p.Leu300Pro | 2 | [ISBT 2012 p214](http://onlinelibrary.wiley.com/doi/10.1111/j.1423-0410.2012.01615_2.x/epdf) | - | 0 | 0.986 | 28 | 0.585 |
|  |  | p.Arg301Leu | 2 | [PMID: 32032242](https://pubmed.ncbi.nlm.nih.gov/32032242/) | - | 0 | 1 | 31 | 0.511 |
|  |  | p.Arg301Cys | 2 | [PMID: 22102705](http://www.ncbi.nlm.nih.gov/pubmed/22102705) | - | 0 | 1 | 32 | 0.871 |
|  |  | p.Arg301His | 2 | [PMID: 22102705](http://www.ncbi.nlm.nih.gov/pubmed/22102705) | - | - | - | 32 | - |
|  |  | p.Arg301Leufs*52 | 3 | [ISBT 2012 p214](http://onlinelibrary.wiley.com/doi/10.1111/j.1423-0410.2012.01615_2.x/epdf) | - | - | - | - | - |
|  |  | p.Thr302Alafs*52 | 3 | [PMID: 23806141](http://www.ncbi.nlm.nih.gov/pubmed/23806141) | - | - | - | - | - |
| Splice site | | c.914-4_914-1delCTAG |  | [PMID: 22102705](http://www.ncbi.nlm.nih.gov/pubmed/22102705) | - | - | - | - | - |
|  |  | c.913+1G>A |  | [PMID: 22102705](http://www.ncbi.nlm.nih.gov/pubmed/22102705) | - | - | - | 34 | - |
| Exon3 |  | p.Glu306Argfs* | 3 | [PMID: 25976964](http://www.ncbi.nlm.nih.gov/pubmed/25976964) | - | - | - | - | - |
|  |  | p.Lys307Asn | 2 | [PMID: 27282573](https://pubmed.ncbi.nlm.nih.gov/27282573/) |  |  |  | 32 |  |
|  | ZF2 | p.Ala310Pro | 2 | [rs145551738](http://www.ncbi.nlm.nih.gov/snp/?term=rs145551738) | - | 0.15 | 0.623 | 27 | 0.097 |
|  |  | p.Trp313Cys | 2 | [PMID: 22102705](http://www.ncbi.nlm.nih.gov/pubmed/22102705) | - | - | - | 33 | - |
|  |  | c.942delA | 2 | [PMID: 30747024](https://pubmed.ncbi.nlm.nih.gov/30747024/) | - | - | - | - | - |
|  |  | p.Cys316Tyr | 2 | [PMID: 25690802](http://www.ncbi.nlm.nih.gov/pubmed/25690802) | - | - | - | 32 | - |
|  |  | p.Cys316* | 3 | [rs572756401](http://www.ncbi.nlm.nih.gov/snp/?term=rs572756401) | 0 | - | - | 36 | - |
|  |  | p.Arg319Glufs*34 | 3 | [PMID: 21821711](http://www.ncbi.nlm.nih.gov/pubmed/21821711) | - | - | - | - | - |
|  |  | p.Arg322Ser | 2 | [rs376711350](http://www.ncbi.nlm.nih.gov/snp/?term=rs376711350) | - | 0 | 0.998 | 34 | 0.297 |
|  |  | p.Ser323Leu | 2 | [ISBT 2012 p214](http://onlinelibrary.wiley.com/doi/10.1111/j.1423-0410.2012.01615_2.x/epdf) | - | - | - | 33 | - |
|  |  | p.Glu325Lys | 4 | [PMID: 21055716](http://www.ncbi.nlm.nih.gov/pubmed/21055716) | - | 0.01 | 1 | 34 | 0.043 |
|  |  | p.Leu326Arg | 2 | [PMID: 21821711](http://www.ncbi.nlm.nih.gov/pubmed/21821711) | - | 0 | 1 | 34 | 0.888 |
|  |  | p.Thr327Ser | 2 | [PMID: 21821711](http://www.ncbi.nlm.nih.gov/pubmed/21821711) | - | 0.01 | 0.999 | 28 | 0.025 |
|  |  | p.Arg328Alafs*31 | 3 | [PMID: 24857170](http://www.ncbi.nlm.nih.gov/pubmed/24857170) | - | - | - | - | - |
|  |  | p.Arg328Leu | 2 | [PMID: 18487511](http://www.ncbi.nlm.nih.gov/pubmed/18487511) | - | - | - | 32 | - |
|  |  | p.Arg328His | 2 | [PMID: 18487511](http://www.ncbi.nlm.nih.gov/pubmed/18487511) | - | 0 | 0.999 | 34 | 0.524 |
|  |  | p.His329Arg | 2 | [PMID: 25976964](http://www.ncbi.nlm.nih.gov/pubmed/25976964) | - | 0 | 1 | 29 | 0.977 |
|  |  | p.Arg331Gly | 2 | [PMID: 18487511](http://www.ncbi.nlm.nih.gov/pubmed/18487511) | - | - | - | 23.8 | - |
|  |  | p.Arg331Trp | 2 | [PMID: 24443441](http://www.ncbi.nlm.nih.gov/pubmed/24443441) | - | - | - | 25.1 | - |
|  |  | p.Lys332Gln | 2 | [PMID: 21273267](http://www.ncbi.nlm.nih.gov/pubmed/21273267) | - | - | - | 31 | - |
|  |  | p.Thr334Lys | 2 | [PMID: 22102705](http://www.ncbi.nlm.nih.gov/pubmed/22102705) | - | - | - | 32 | - |
|  |  | p.Thr334Arg | 2 | [PMID: 22102705](http://www.ncbi.nlm.nih.gov/pubmed/22102705) | - | 0 | 1 | 32 | 0.482 |
|  |  | p.Gly335Arg | 2 | [PMID: 24443441](http://www.ncbi.nlm.nih.gov/pubmed/24443441) | - | 0 | 1 | 32 | 0.657 |
|  |  | p.Pro338Thr | 2 | [PMID: 24857170](http://www.ncbi.nlm.nih.gov/pubmed/24857170) | - | 0 | 1 | 32 | 0.631 |
|  |  | p.Pro338Ser | 2 | [PMID: 24930900](http://www.ncbi.nlm.nih.gov/pubmed/24930900) | - | 0 | 1 | 33 | 0.695 |
|  | ZF3 | p.Cys341Tyr | 2 | [PMID: 24829204](http://www.ncbi.nlm.nih.gov/pubmed/24829204) | - | 0 | 1 | 34 | 0.975 |
|  |  | p.Gln342His | 2 | [rs146658904](http://www.ncbi.nlm.nih.gov/snp/?term=rs146658904) | 0 | 0.48 | 0.062 | 22 | 0.211 |
|  |  | p.Pro345Leu | 2 | [rs370574267](http://www.ncbi.nlm.nih.gov/snp/?term=rs370574267) | - | 0.01 | 0.21 | 25 | 0.214 |
|  |  | p.His357Pro | 2 | [PMID: 25976964](http://www.ncbi.nlm.nih.gov/pubmed/25976964) | - | 0 | 0.999 | 29 | 0.996 |
|  |  | p.His357Gln | 2 | [PMID: 23125034](http://www.ncbi.nlm.nih.gov/pubmed/23125034) | - | 0 | 0.999 | 27 | 0.996 |
|  |  | p.Met358Ile | 2 | [PMID: 27282573](https://pubmed.ncbi.nlm.nih.gov/27282573/) | - | - | - | 29.7 | - |
|  |  | p.Arg360His | 2 | [PMID: 27821015](https://pubmed.ncbi.nlm.nih.gov/27821015/) | - | 0 | 0.998 | 29 | 0.318 |

# Abbreviations: MAF, Minor Allele Frequency; CHS, Southern Han Chinese.

# ^a^Data of MAF is from The 1000 Genomes Project (phase3).

# ^b^The pathogenicity was tested by bioinformatics software SIFT, PolyPhen, Combined Annotation Dependent Depletion(CADD) and Mutation Assessor.

**Supplemental Table 2.** The Hb level of *KLF1* mutation carriers in references

| **Patient** | ***KLF1* genotype** | | ***KLF1* classification** | | **Hb(g/L)** | **Reference** |
| --- | --- | --- | --- | --- | --- | --- |
|  | **Allele1** | **Allele2** | **Allele1** | **Allele2** |  |  |
| 1 | -154C>T | N | P | N | 116 | [PMID: 24443441](https://pubmed.ncbi.nlm.nih.gov/24443441/) |
| 2 | Gly211Ala | N | 1 | N | 76 | [PMID: 28369821](https://pubmed.ncbi.nlm.nih.gov/28369821/) |
| 3 | Gly211Ala | N | 1 | N | 90 | [PMID: 28369821](https://pubmed.ncbi.nlm.nih.gov/28369821/) |
| 4 | Gly335Arg | N | 2 | N | 134 | [PMID: 23806141](https://pubmed.ncbi.nlm.nih.gov/23806141/) |
| 5 | Arg331Trp | N | 2 | N | 122 | [PMID: 24443441](https://pubmed.ncbi.nlm.nih.gov/24443441/) |
| 6 | Arg301His | N | 2 | N | 156 | [PMID: 24443441](https://pubmed.ncbi.nlm.nih.gov/24443441/) |
| 7 | Ala298Pro | N | 2 | N | 140 | [PMID: 24443441](https://pubmed.ncbi.nlm.nih.gov/24443441/) |
| 8 | Ala298Pro | N | 2 | N | 121 | [PMID: 24443441](https://pubmed.ncbi.nlm.nih.gov/24443441/) |
| 9 | Ala298Pro | N | 2 | N | 144 | [PMID: 24443441](https://pubmed.ncbi.nlm.nih.gov/24443441/) |
| 10 | Ala298Pro | N | 2 | N | 132 | [PMID: 24443441](https://pubmed.ncbi.nlm.nih.gov/24443441/) |
| 11 | Ala298Pro | N | 2 | N | 117 | [PMID: 24443441](https://pubmed.ncbi.nlm.nih.gov/24443441/) |
| 12 | Ala298Pro | N | 2 | N | 135 | [PMID: 24443441](https://pubmed.ncbi.nlm.nih.gov/24443441/) |
| 13 | Ala298Pro | N | 2 | N | 115 | [PMID: 24857170](https://pubmed.ncbi.nlm.nih.gov/24857170/) |
| 14 | His299Asp | N | 2 | N | 134 | [PMID: 24857170](https://pubmed.ncbi.nlm.nih.gov/24857170/) |
| 15 | Ser270Trp | N | 2 | N | 104 | [PMID: 24857170](https://pubmed.ncbi.nlm.nih.gov/24857170/) |
| 16 | Thr334Arg | N | 2 | N | 100 | [PMID: 24857170](https://pubmed.ncbi.nlm.nih.gov/24857170/) |
| 17 | Pro338Thr | N | 2 | N | 115 | [PMID: 24857170](https://pubmed.ncbi.nlm.nih.gov/24857170/) |
| 18 | Cys341Tyr | N | 2 | N | 114 | [PMID: 24857170](https://pubmed.ncbi.nlm.nih.gov/24857170/) |
| 19 | Cys316Tyr | N | 2 | N | 132 | [PMID: 25690802](https://pubmed.ncbi.nlm.nih.gov/25690802/) |
| 20 | Arg360His | N | 2 | N | 80 | [PMID: 27821015](https://pubmed.ncbi.nlm.nih.gov/27821015/) |
| 21 | Arg301Cys | N | 2 | N | 127 | [PMID: 22102705](https://pubmed.ncbi.nlm.nih.gov/22102705/) |
| 22 | Arg301His | N | 2 | N | 106 | [PMID: 22102705](https://pubmed.ncbi.nlm.nih.gov/22102705/) |
| 23 | Trp313Cys | N | 2 | N | 121 | [PMID: 22102705](https://pubmed.ncbi.nlm.nih.gov/22102705/) |
| 24 | Thr334Lys | N | 2 | N | 107 | [PMID: 22102705](https://pubmed.ncbi.nlm.nih.gov/22102705/) |
| 25 | Lys332Gln | N | 2 | N | 123 | [PMID: 21273267](https://pubmed.ncbi.nlm.nih.gov/21273267/) |
| 26 | Lys332Gln | N | 2 | N | 154 | [PMID: 21273267](https://pubmed.ncbi.nlm.nih.gov/21273267/) |
| 27 | Gly176Argfs*179 | N | 3 | N | 104 | [PMID: 23806141](https://pubmed.ncbi.nlm.nih.gov/23806141/) |
| 28 | Gly176Argfs*179 | N | 3 | N | 137 | [PMID: 23806141](https://pubmed.ncbi.nlm.nih.gov/23806141/) |
| 29 | Gly176Argfs*179 | N | 3 | N | 131 | [PMID: 23806141](https://pubmed.ncbi.nlm.nih.gov/23806141/) |
| 30 | Gly176Argfs*179 | N | 3 | N | 138 | [PMID: 23806141](https://pubmed.ncbi.nlm.nih.gov/23806141/) |
| 31 | Thr302Alafs*52 | N | 3 | N | 129 | [PMID: 23806141](https://pubmed.ncbi.nlm.nih.gov/23806141/) |
| 32 | Gly176Argfs*179 | N | 3 | N | 114 | [PMID: 24443441](https://pubmed.ncbi.nlm.nih.gov/24443441/) |
| 33 | Gly176Argfs*179 | N | 3 | N | 125 | [PMID: 24443441](https://pubmed.ncbi.nlm.nih.gov/24443441/) |
| 34 | Gln58* | N | 3 | N | 140 | [PMID: 24443441](https://pubmed.ncbi.nlm.nih.gov/24443441/) |
| 35 | Gly176Argfs*179 | N | 3 | N | 141 | [PMID: 24443441](https://pubmed.ncbi.nlm.nih.gov/24443441/) |
| 36 | Gly176Argfs*179 | N | 3 | N | 139 | [PMID: 24443441](https://pubmed.ncbi.nlm.nih.gov/24443441/) |
| 37 | Gly176Argfs*179 | N | 3 | N | 118 | [PMID: 24443441](https://pubmed.ncbi.nlm.nih.gov/24443441/) |
| 38 | Gly176Argfs*179 | N | 3 | N | 119 | [PMID: 24443441](https://pubmed.ncbi.nlm.nih.gov/24443441/) |
| 39 | Gly176Argfs*179 | N | 3 | N | 127 | [PMID: 24443441](https://pubmed.ncbi.nlm.nih.gov/24443441/) |
| 40 | Arg319Glufs*34 | N | 3 | N | 120 | [PMID: 25724378](https://pubmed.ncbi.nlm.nih.gov/25724378/) |
| 41 | Trp30* | N | 3 | N | 164 | [PMID: 25724378](https://pubmed.ncbi.nlm.nih.gov/25724378/) |
| 42 | Ser270* | N | 3 | N | 146 | [PMID: 22093801](asmart.com.cn/product-details/page/3853/379138097) |
| 43 | Ser270* | N | 3 | N | 139 | [PMID: 22093801](asmart.com.cn/product-details/page/3853/379138097) |
| 44 | Ser270* | N | 3 | N | 121 | [PMID: 22093801](asmart.com.cn/product-details/page/3853/379138097) |
| 45 | Ser270* | N | 3 | N | 126 | [PMID: 22093801](asmart.com.cn/product-details/page/3853/379138097) |
| 46 | Gly176Argfs*179 | N | 3 | N | 130.4 | [PMID: 24857170](https://pubmed.ncbi.nlm.nih.gov/24857170/) |
| 47 | Gly176Argfs*179 | N | 3 | N | 144 | [PMID: 29393578](https://pubmed.ncbi.nlm.nih.gov/29393578/) |
| 48 | Gly176Argfs*179 | N | 3 | N | 135 | [PMID: 29393578](https://pubmed.ncbi.nlm.nih.gov/29393578/) |
| 49 | Gly176Argfs*179 | N | 3 | N | 141 | [PMID: 29393578](https://pubmed.ncbi.nlm.nih.gov/29393578/) |
| 50 | Gly176Argfs*179 | N | 3 | N | 136 | [PMID: 29393578](https://pubmed.ncbi.nlm.nih.gov/29393578/) |
| 51 | Gly176Argfs*179 | N | 3 | N | 146 | [PMID: 29393578](https://pubmed.ncbi.nlm.nih.gov/29393578/) |
| 52 | Gly176Argfs*179 | N | 3 | N | 89 | [PMID: 31111750](https://pubmed.ncbi.nlm.nih.gov/31111750/) |
| 53 | Gly176Argfs*179 | N | 3 | N | 113 | [PMID: 31111750](https://pubmed.ncbi.nlm.nih.gov/31111750/) |
| 54 | Lys54Profs*9 | N | 3 | N | 143 | [PMID: 22102705](https://pubmed.ncbi.nlm.nih.gov/22102705/) |
| 55 | Arg328H* | N | 3 | N | 126 | [PMID: 22102705](https://pubmed.ncbi.nlm.nih.gov/22102705/) |
| 56 | Arg328L* | N | 3 | N | 130 | [PMID: 22102705](https://pubmed.ncbi.nlm.nih.gov/22102705/) |
| 57 | Gly176Argfs*179 | N | 3 | N | 137 | [PMID: 22102705](https://pubmed.ncbi.nlm.nih.gov/22102705/) |
| 58 | Gly176Argfs*179 | N | 3 | N | 138 | [PMID: 22102705](https://pubmed.ncbi.nlm.nih.gov/22102705/) |
| 59 | Ser270* | N | 3 | N | 129 | [PMID: 21273267](https://pubmed.ncbi.nlm.nih.gov/21273267/) |
| 60 | Glu325Lys | N | 4 | N | 80 | [PMID: 23522491](https://pubmed.ncbi.nlm.nih.gov/23522491/) |
| 61 | Glu325Lys | N | 4 | N | 63 | [PMID: 21055716](https://pubmed.ncbi.nlm.nih.gov/21055716/) |
| 62 | Glu325Lys | N | 4 | N | 80 | [PMID: 21778342](https://pubmed.ncbi.nlm.nih.gov/21778342/) |
| 63 | -148G>A | Ser102Pro | P | 1 | 128 | [PMID: 23161389](https://pubmed.ncbi.nlm.nih.gov/23161389/) |
| 64 | ­154C>T | Ala298Pro | P | 2 | 77 | [PMID: 24443441](https://pubmed.ncbi.nlm.nih.gov/24443441/) |
| 65 | Gly335Arg | Arg331Trp | 2 | 2 | 30 | [PMID: 24443441](https://pubmed.ncbi.nlm.nih.gov/24443441/) |
| 66 | Arg301Cys | Arg301Cys | 2 | 2 | 121 | [PMID: 29980343](https://pubmed.ncbi.nlm.nih.gov/29980343/) |
| 67 | Gly176Argfs*179 | Arg301His | 3 | 2 | 75 | [PMID: 24443441](https://pubmed.ncbi.nlm.nih.gov/24443441/) |
| 68 | Gln58* | Ala298Pro | 3 | 2 | 66 | [PMID: 24443441](https://pubmed.ncbi.nlm.nih.gov/24443441/) |
| 69 | Gly176Argfs*179 | Ala298Pro | 3 | 2 | 34 | [PMID: 24443441](https://pubmed.ncbi.nlm.nih.gov/24443441/) |
| 70 | Gly176Argfs*179 | Ala298Pro | 3 | 2 | 83 | [PMID: 24443441](https://pubmed.ncbi.nlm.nih.gov/24443441/) |
| 71 | Gly176Argfs*179 | Ala298Pro | 3 | 2 | 57 | [PMID: 24443441](https://pubmed.ncbi.nlm.nih.gov/24443441/) |
| 72 | Gly176Argfs*179 | Ala298Pro | 3 | 2 | 54 | [PMID: 24443441](https://pubmed.ncbi.nlm.nih.gov/24443441/) |
| 73 | Ser270* | Lys332Gln | 3 | 2 | 115 | [PMID: 21273267](https://pubmed.ncbi.nlm.nih.gov/21273267/) |
| 74 | Ser270* | Lys332Gln | 3 | 2 | 121 | [PMID: 21273267](https://pubmed.ncbi.nlm.nih.gov/21273267/) |
| 75 | Gly176Argfs*179 | Ala298Pro | 3 | 2 | 79 | [PMID: 25585695](https://pubmed.ncbi.nlm.nih.gov/25585695/) |
| 76 | Gly176Argfs*179 | Pro338Ser | 3 | 2 | 97 | [PMID: 25585695](https://pubmed.ncbi.nlm.nih.gov/25585695/) |
| 77 | His295Leufs*58 | Arg301Leu | 3 | 2 | 61 | [PMID: 32032242](https://pubmed.ncbi.nlm.nih.gov/32032242/) |
| 78 | Gly176Argfs*179 | Pro338Thr | 3 | 2 | 30 | [PMID: 28361594](https://pubmed.ncbi.nlm.nih.gov/28361594/) |
| 79 | Gly176Argfs*179 | Pro338Thr | 3 | 2 | 67 | [PMID: 31645145](https://pubmed.ncbi.nlm.nih.gov/31645145/) |
| 80 | Trp30* | Arg319Glufs*34 | 3 | 3 | 65 | [PMID: 25724378](https://pubmed.ncbi.nlm.nih.gov/25724378/) |
